# Arginine methylation regulates Ewing sarcoma cell viability in a *EWSR1::FLI1* dependent manner and provides a therapeutic opportunity

**DOI:** 10.3389/fonc.2025.1538208

**Published:** 2025-08-01

**Authors:** Ciara M. Ward, Charles Brockwell, Gavin S. McNee, Emily Orton, Emily N. P. Prowse, Susanne A. Gatz, Clare C. Davies

**Affiliations:** ^1^ Department of Cancer and Genomic Sciences, College of Medical and Health Sciences, University of Birmingham, Birmingham, United Kingdom; ^2^ School of Life Sciences, University of Wolverhampton, Wolverhampton, United Kingdom; ^3^ Department of Paediatric Oncology, Birmingham Women’s and Children’s NHS Foundation Trust, Birmingham, United Kingdom

**Keywords:** Ewing sarcoma, PRMT1, PRMT5, arginine methylation, DNA damage, olaparib, therapeutics

## Abstract

**Introduction:**

Ewing sarcoma is a rare type of cancer arising from bone and soft tissues mainly affecting children and young adults. Treatments include intensive chemotherapy, surgery and radiotherapy, however more than 30% of patients die from the disease. Direct drug targeting of EWS-FLI1 remains a significant challenge, therefore new approaches are urgently required.

**Methods:**

Analysis of *PRMT1* and *PRMT5* transcript expression using the R2 platform focusing on the Filion dataset of sarcomas that includes Ewing sarcoma patients alongside other fusion-positive sarcomas and breast and lung cancer datasets. Immunoblotting across a panel of Ewing sarcoma cell lines detected PRMT1 and PRMT5 expression and associated activity. Cell viability assay after PRMT inhibition, with and without olaparib, were conducted by trypan blue exclusion and MTT assay. DNA damage was detected by immunofluorescence staining for markers of DNA damage (γH2AX) and double-strand breaks (53BP1).

**Results:**

We show that the expression and activity of the arginine methyltransferases PRMT1 and PRMT5 are elevated in Ewing sarcoma and that inhibition of PRMT1 or PRMT5 with pre-clinical inhibitors leads to growth arrest and apoptosis that is dependent on the expression of the driver oncogene *EWSR1::FLI1.* Mechanically, we show that PRMT1 and PRMT5 inhibitors promote DNA damage, and that PRMT5 inhibitors synergise with the PARP inhibitor olaparib to induce elevated DNA damage and reduced cell viability.

**Discussion:**

Our study implies that PRMT1/PRMT5 are important mediators of *EWSR1::FLI1* oncogenicity and that drug targeting PRMT1/PRMT5 in combination with DNA damaging chemotherapies could be an effective therapeutic strategy for the treatment of ES patients.

## Introduction

Ewing Sarcoma (ES) is a rare cancer of bone and soft tissues affecting children and young adults. Standard of care involves multimodal therapy with non-specific chemotherapies, radiotherapy and surgery. Despite this intensive regime, those with metastatic or recurrent disease have a particularly poor outcome and 30% of patients are not cured (1, 2). New therapeutic approaches are thus urgently required to provide better, safer, and kinder treatment options.

ES are characterised by distinct chromosomal translocations that generate a gene fusion of a member of the RNA binding FET gene family (*EWSR1, FUS*) and a member of the ETS transcription factor gene family (*FLI1, ERG, FEV, ETV1 and ETV4*). The most common genetic event occurring in 85%-90% of cases is the production of the *EWSR1::FLI1* gene fusion encoding the EWS-FLI1 fusion protein that functions as an aberrant transcription factor (1, 3). This oncogenic fusion protein has been the focus of translational research for many years, but direct targeting has remained elusive for imminent clinical application. Therapeutic avenues have thus tried to identify vulnerabilities and dependencies created through the fusion protein itself. For example, the EWS-FLI1 fusion protein promotes the rewiring of transcriptional programs, the generation of oncogenic alternative splicing products (4, 5), and genome instability through the sequestering of BRCA1 leading to aberrant R-loop formation and replication stress (6, 7). Consequently, ES cells display sensitivity to PARP inhibitors attributed to defective homologous recombination (HR) (6). Despite this, efforts to translate these findings to the clinic have faced challenges. Treatment with PARP inhibitors as a single agent showed no clinical benefit (8, 9), and whilst chemotherapy/PARP inhibitor combinations promoted responses in a few patients, combination drug doses were limited due to toxicity (10–12). Together, this implies that the underlying oncogenic mechanisms of ES are complex and that combining PARP inhibition with chemotherapy is not sufficient to create a therapeutic window that would allow ES cells to be preferentially sensitised over healthy patient cells.

Protein post-translational modifications are vital for increasing the functional diversity of the proteome, however their deregulation promotes cancer initiation and progression. Methylation of arginine residues by protein arginine methyltransferases (PRMTs) is one such modification with elevated expression of PRMTs highly associated with the development, pathogenesis, and drug resistance of adult solid and haematological cancers (13). PRMTs catalyse mono- and dimethylation of the guanidino group of the arginine residue using S-adenosyl methionine (SAM) as a methyl donor. Dimethylation can occur asymmetrically (ADMA) by Type 1 enzymes (PRMT1/PRMT2/PRMT3/CARM1/PRMT6/PRMT8) where two methyl groups are placed onto one of the terminal nitrogen atoms of the guanidino group, or symmetrically (SDMA) by Type II enzymes (PRMT5/PRMT9) where one methyl group is placed onto each of the terminal nitrogen groups (14). PRMT1 and PRMT5 are the predominant mediators of ADMA and SDMA respectively and are known to methylate histone and non-histone proteins. This contributes to a wide range of cellular processes that are deregulated in cancer, including cancer cell survival, stemness, migration, immune invasion, genome stability and chemoresistance (13, 14). Mechanistically, PRMT1/PRMT5 regulate oncogenic epigenetic-mediated gene expression, mRNA splicing, DNA repair, epithelial-mesenchymal transition, and receptor signalling (13). As such, genetic or siRNA-mediated depletion of PRMT1 or PRMT5 levels result in decreased cancer cell proliferation (15–17) and co-operate with DNA damaging chemotherapies to reduce cell viability (18–21). The development of clinically relevant inhibitors for specific PRMTs, particularly for PRMT5, has thus progressed at a phenomenal rate with several Phase I/II clinical trials initiated for adult haematological, breast and lung cancer (14, 22).

Intriguingly, many of the functions of the EWS-FLI1 fusion protein are shared with those identified for PRMTs. For example, EWS is an arginine methylated protein (23), and both PRMT1 and PRMT5 regulate R-loop formation and DNA repair via HR (18, 24–26). PRMTs are also key regulators of canonical and alternative splicing (13), whilst arginine methylation regulates phase separation (27), an inherent property of the FET family of RNA binding proteins. In this work, we show that PRMT1/PRMT5 expression and associated ADMA/SDMA is elevated in ES cells. Inhibition of PRMT1 or PRMT5 leads to reduced ES cell proliferation, whilst combining both PRMT inhibitors leads to a robust induction of apoptosis. Mechanistically, PRMT inhibitors promote DNA damage and potentiate the effects of olaparib thereby reducing ES cell survival. Critically, the cell-detrimental effect of PRMT inhibition was dependent on the expression of EWS-FLI1 implying a therapeutic window of opportunity for the clinical use of PRMT inhibitors. Taken together, our study implies that PRMT1/PRMT5 are important mediators of EWS-FLI1 oncogenicity and that drug targeting of PRMT1/PRMT5 in combination with DNA damaging chemotherapies could be an effective therapeutic strategy for the treatment of ES patients.

## Materials and methods

### Cell lines and cell culture

TC-32 Ewing sarcoma cell line was maintained in RPMI 1640 supplemented with 10% foetal bovine serum (FBS), 1% L-glutamine and 1% penicillin and streptomycin. SK-N-MC Ewing sarcoma cell line was maintained in Dulbecco’s Modified Eagle Medium (DMEM)/F12 (Ham) (1:1)) supplemented with 10% FBS, 1% glutamine, 1% penicillin and streptomycin. A673 (ATCC) and A673 clone ASP#14 (28) (herein referred to as A673-tetON-shEWSR1::FLI1) Ewing sarcoma cell lines were maintained in DMEM supplemented with 10% FBS, 1% Glutamine, 1% penicillin and streptomycin. A673-tetON-shEWSR1::FLI1 were maintained in 50µg/ml Zeocin and 2μg/ml Blasticidin. BMMSC (bone marrow mesenchymal stem cells) were a gift from Helen McGettrick (University of Birmingham) and were maintained in mesenchymal stem cell growth medium 2 (Promocell) in penicillin/streptomycin-free conditions. HFF1 cells were a gift from Joanna Parish (University of Birmingham) and cultured in DMEM supplemented with 10% FBS, 1% glutamine, 1% penicillin and streptomycin. The siRNA sequences are: siCTRL: 5’-CGUACGCGGAAUACUUCGA-3’; siPRMT5: 5’-CGAAAAGCUG ACACACUA-3’; siPRMT1: 5’-UGAGCGUUCCUAGGCGGUUUC-3’. SK-N-MC-TetON-shPRMT1 and SK-N-MC-TetON-shPRMT5 cell lines were generated by lentiviral infection of pTRIPZ-shPRMT1 (construct ID: V3THS-381699; Horizon Discovery) and pLKO-tetOn-shPRMT5 constructs. Sequences are: shPRMT1 (5’-TCATTGCAGGCAGAACGG-3’); shPRMT5-2 (5’-AGGGACTGGAATACGCTAATT-3’) (16).

### Drug treatments

Please see associated figure legends for concentrations and durations of GSK591 (PRMT5 inhibitor) (29, 30), MS023 (Type 1 PRMT inhibitor) (31) and olaparib treatments.

### Trypan blue cell viability assays

Cells were plated in duplicate in 6 well plate and treated with GSK591 and/or MS023 for 4 days at the dose indicated in the figure legend. For trypan blue exclusion assay, media was removed, and cells resuspended in trypan blue solution. Cell numbers were determined using the Cellometer cell counter (Nexcelom Bioscience) and the Cellometer Auto Counter software (Version 3.3.5.11, Nexcelom Bioscience). For A673-tetON-shEWSR1::FLI1 cells, cells were plated in duplicate in 6 well plate and treated with 2μg/ml doxycycline and/or 1μM olaparib as required. 72 hrs later, media was replaced and GSK591/MS023 included as desired. Cells were harvested for trypan blue exclusion assay 4 days later. SK-N-MC-TetON-shPRMT1 and SK-N-MC-TetON-shPRMT5 cell lines were treated with 1μg/ml doxycycline for 7 days and viable cell number determined by trypan blue exclusion analysis.

### MTT assay

Cells were plated in triplicate in 96 well plate and treated with GSK591 and DNA damaging agents for 6 days at the dose indicated in the figure legend. MTT (0.5mg/ml) was added for 2 hours, solubilised by the addition of acidified isopropanol and absorbance measure at 560nm. Data presented is normalised to samples that did not receive DNA damaging agents.

### Transcriptomic analysis

Data mining of the R2 platform for *PRMT5*, *MEP50* and *PRMT1* expression was accessed via https://hgserver1.amc.nl/cgi-bin/r2/main.cgi?open_page=login using platforms MAS 5.0-u133a and MAS 5.0 u133p2. Probe set were: *PRMT5 =* 217786_s_at; *MEP50/WDR77 =* 201421_s_at; *PRMT1 =* 206445_s_at). The following datasets were accessed: Filion (n=137; 7 different fusion positive sarcoma subtypes including n=24 *EWSR1-FLI1* and n=4 *EWSR1-ERG*) (32), breast cancer sets Miller et al. (n=251) and Sinn et al. (n=1108), and lung cancer sets Jen et al. (n=107).The Ewing Sarcoma Cell Line Atlas (ESCLA) contains whole genome DNA methylation, transcriptome, proteome and chromatin immunoprecipitation sequencing data of 18 cell lines with doxycycline (dox)-inducible knockdown of the respective fusion protein (33). Data presented as log2-transformed.

### Immunocytochemistry

Cells were trypsinised and washed three times with ice cold PBS before fixing in ice-cold 4% (w/v) paraformaldehyde (Merck Life Sciences) for 12 mins. Following three washes with ice-cold PBS, 100,000 cells were centrifuged (5 min) onto a 6mm-diameter circular area on microscopic slides using a Shandon CytoSpin cytocentrifuge (Thermo Scientific). Slides were allowed to air dry for 15 min before treatment with ice-cold cytoskeletal pre-extraction buffer (PBS-0.5% Triton X-100 containing 10mM PIPES (pH 6.0), 300mM Sucrose, 100mM NaCl and 3mM MgCl_2_) for 10 min at room temperature. After two rinses with ice-cold PBS, cells were permeabilised with PBS-0.5% (v/v) Triton X-100 for 5 min at room temperature, washed with ice-cold PBS (3 × 5 min), and blocked with PBS/5% FBS/1% bovine serum albumin/0.3% Triton X-100 for 1 hr at room temperature. Cells were incubated in a humid chamber at 4°C overnight with the primary antibody diluted in blocking buffer. Slides were washed three times with PBS-0.1% Tween (5 min), incubated with the relevant fluorophore-conjugated secondary antibody diluted with blocking buffer for 1 hr at room temperature in the dark, washed three times with PBS-0.1% Tween (5 min) and nuclei counterstained using ProLong Gold Antifade mounting media containing 4′,6-diamidino-2-phenylindole (DAPI) (Invitrogen). For detection of 53BP1 foci in EdU+ cells, EdU (10μM) was added 30 mins to cell culture media before cells were trypsinised, fixed in 3.6% PFA and collected for cytospin (as to protocol above). After pre-extraction (PBS-0.5% Triton X-100 containing 10mM PIPES (pH 6.0), 300mM Sucrose, 100mM NaCl and 3mM MgCl_2_; 10 min at room temperature), slides were incubated in blocking solution (5% FBS in PBS-0.3% Triton X-100) for 5 mins and Click-IT reaction (4mM CuSO4, 100mM sodium ascorbate, 1:800 Alexa-Fluro 594 Azide (Thermo Fisher), in x1 Tris-buffer saline (TBS)) for 30 mins at room temperature in the dark. Following three washes (5 mins) with PBS-0.3% (v/v) Triton X-100, slides were blocked in with PBS/5% FBS/1% bovine serum albumin/0.3% Triton X-100 for 1 hr at room temperature, incubated with primary antibody overnight, followed by three PBS-0.1% Tween washes (5 min) and incubation with the relevant fluorophore-conjugated secondary antibody diluted with blocking buffer for 1 hr at room temperature in the dark. After three washed with PBS-0.1% Tween (5 mins), nuclei counterstained using ProLong Gold Antifade mounting media containing 4′,6-diamidino-2-phenylindole (DAPI) (Invitrogen). 53BP1 and Rad51 foci number was counted by eye using a Zeiss AxioVert 3 microscope; γH2AX intensity was determined by analysis >100 nuclei per experimental replicate using ImageJ software. Antibodies used for immunostaining were: γH2AX (1:1000; Millipore; #05-636); 53BP1 (1:1000; Novas Biologicals; #NB100-304); Rad51 (1:500; Millipore; PC130); DyLight 488 anti-Rabbit (1:1000; Invitrogen; #35552) and Alexa Fluor 594 anti-Mouse (1:1000; Invitrogen; #A11032).

### Immunoblotting

Cell lysates were prepared by sonicating in ice-cold UTB buffer (8M Urea, 50mM Tris, pH 7.5, 150mM 2-mercaptoethanol), sonicated twice at 25% amplitude using a thin probe (5 seconds), and clarified by centrifugation. Proteins were separated by SDS-PAGE and transferred to PVDF membrane, blocked and incubated overnight in primary antibody. Membranes were washed and incubated with secondary antibody and visualised by enhanced chemiluminescence and X-ray film. Antibodies used for immunoblotting were: PRMT1 (1:1000; Cell Signalling Technology; #2449); PRMT5 (1:1000; Active Motif; #61001); MEP50 (1:1000; Cell Signalling Technology; #2823); ADMA (1:1000; Epicypher; #13281001); ADMA (1:1000; Cell Signalling Technology; #13222); MMA (1:1000; Cell Signalling Technology; #8015); FLI1 (1:1000; BD Pharmingen; #554266); phospho-RPA-Ser4/8 (1:1000; Bethyl; #A300-245A); RPA (1:1000; Calbiochem; #NA18); phospho-Chk1-Ser345 (1:1000; Cell Signalling Technology; #2348); Chk1 (1:1000; Cell Signalling Technology; #2360); β-actin-HRP (1:5000; Cell Signalling Technology; #5125); GAPDH (1:5000; Cell Signalling Technology; #2118); γH2AX (1:1000; Millipore; #05-636); cleaved caspase-3-Asp175 (1:1000; Cell Signalling Technology; #9661); Rabbit IgG-HRP (1:5000; DAKO; #P0399); Mouse IgG-HRP; 1:5000; Cell Signalling Technology; #7076).

### Cell cycle analysis

Cells were treated with GSK591/MS023 for 48 hrs and pulsed for 60 mins with 50μM BrdU before trypsinisation and collection. Cells were washed in ice-cold PBS and fixed in 70% EtOH. For BrdU staining, cells were washed twice with ice-cold PBS and resuspended in 2M HCl for 20 mins at room temperature. Cells were washed twice with ice-cold PBS and once with ice-cold PBS-0.2% Tween-20/1% BSA (PBS-T20-BSA). Cells were incubated with 20μl anti-BrdU-FITC (BD Biosciences) in a total volume of 100μl of PBS-T20-BSA and incubated in the dark on ice for 1 hr. Following two washes with PBS-T20-BSA and one wash with PBS, cells were resuspended in PBS (25μg/ml propidium iodide/100μg/ml RNaseA) and analysed by flow cytometry using a BD LSR Fortessa X-20 and FlowJo software.

### Statistical analysis

The means of two independent groups were compared by a Student’s *t*-test (two-sided, equal variance). Results are expressed as mean ± SEM or SD, and data were analysed by GraphPad Prism 9.2.0 and the Excel program of Microsoft Office.

## Results

### PRMT1, PRMT5 and associated enzymatic activities are elevated in ES cells

**Figure 1:**
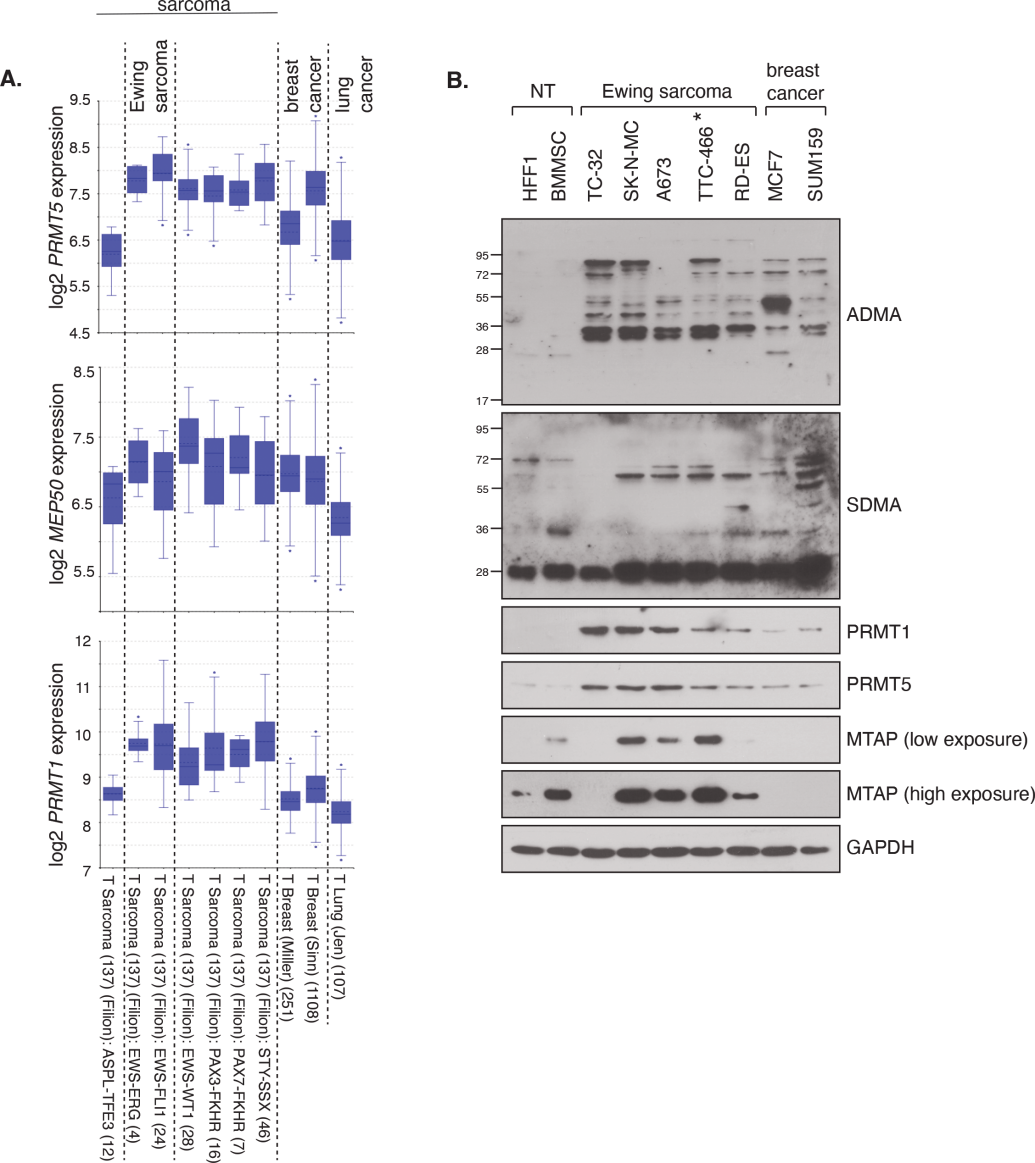
PRMT1 and PRMT5 expression and activity is elevated in Ewing sarcoma. **(A)** mRNA expression levels of *PRMT1*, *PRMT5* and *MEP50* in fusion-positive sarcomas, including Ewing sarcoma (*EWRS1::FLI1* and *EWSR1::ERG*; dataset 2 and 3), in comparison to breast and lung cancer tissues. Data taken from the R2 platform of Affymetrix datasets (MAS 5.0-u133a - *PRMT5* 217786_s_at; *MEP50/WDR77* 201421_s_at; *PRMT1* 206445_s_at). **(B)** Protein levels of PRMT1 and PRMT5, and associated activity, as determined by ADMA and SDMA levels, is elevated in a panel of ES cell lines compared to non-transformed (NT) HFF1 and BMMSC cells. Data representative of n=3 biologically independent experiments. Asterisk indicates EWSR1::ERG fusion cell line.

As a first indicator for a potential relevance of PRMT1 and PRMT5 in ES, we queried the R2 platform for PRMT1 and PRMT5 mRNA expression focusing on the Filion dataset which includes ES alongside other fusion-positive sarcomas (32). We included two breast and one lung cancer dataset for comparison as these tumour types are the focus of PRMT5 inhibitor phase I/II trials for adult cancers (14, 22). We found that *PRMT1* and *PRMT5* expression, along with *MEP50* the obligate cofactor of PRMT5, are generally higher in multiple sarcoma types, including *EWSR1::FLI1* and *EWSR1::ERG* fusion-positive ES tumours, than in breast and lung cancer (**Figure 1A**, **Supplementary Figure 1A**). Screening of a panel of four *EWSR1::FLI1* and one *ESWR1::ERG* fusion ES cell lines supported the transcriptional profiling, with elevated PRMT1 and PRMT5 protein expression and SDMA and ADMA levels compared to non-transformed human foreskin fibroblast cells (HFF1) and bone marrow mesenchymal stem cells [BMMSC - the perceived cell of origin of ES (34)] (**Figure 1B**). Again, protein levels of PRMT1/PRMT5 were generally higher in ES cells compared to MCF7 and SUM159 breast cancer lines (**Figure 1B**). PRMT1, PRMT5, SDMA and ADMA are thus elevated in ES cells compared to normal tissue.

### PRMT1 and PRMT5 regulate the growth of *EWSR1::FLI1* fusion ES cells

**Figure 2:**
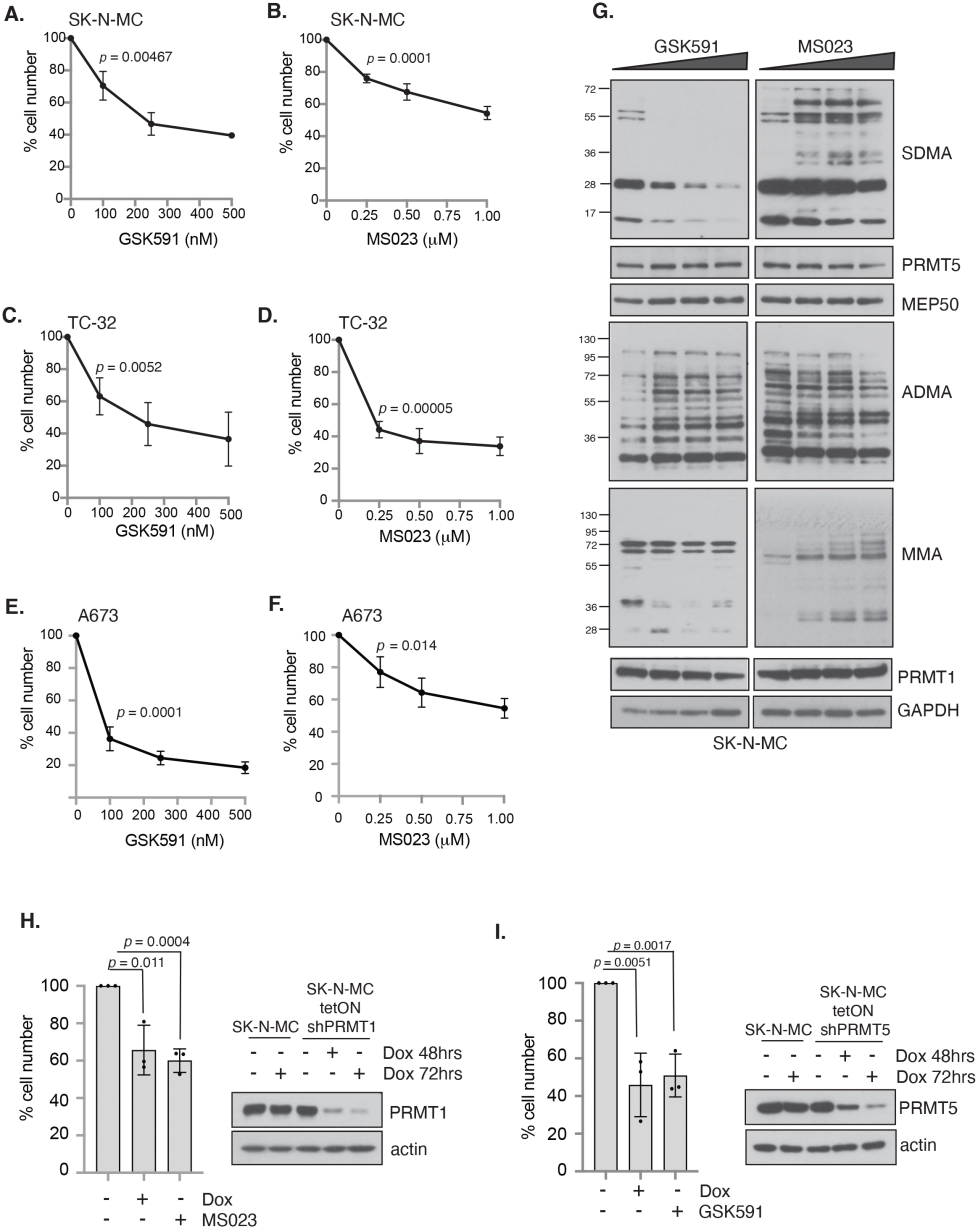
Inhibition of PRMT activity reduces cell viability in a panel of Ewing sarcoma cell lines. Cells were treated with either the PRMT5 inhibitor GSK591 or the Type I PRMT inhibitor MS023 for 4 days and viability measured by trypan blue exclusion. **(A, B)** SK-N-MC; **(C, D)** TC-32; **(E, F)** A673. n=3; mean ± SD; Student’s *t*-test (two-sided, equal variance). Statistics carried on lowest dose of GSK591 or MS023 compared to untreated. **(G)** GSK591 and MS023 alters levels of ADMA, SDMA and MMA in SK-N-MC cells. Data representative of n=3 biologically independent experiments. **(H)** Inducible knockdown of PRMT1 in SK-N-MC cells reduced cell viability to a similar extent as MS023 treatment. Cells were treated with doxycycline (1μg/ml) for 7 days and MS023 (1μM) for 4 days starting 72 hours after start of doxycycline treatment, and viability assessed by trypan blue exclusion. n=3; mean ± SD; Student’s *t*-test (two-sided, equal variance). **(I)** Inducible knockdown of PRMT5 in SK-N-MC cells reduced cell viability to a similar extent as GSK591 treatment. Cells were treated with doxycycline (1μg/ml) for 7 days and GSK591 (500nM) for 4 days starting 72 hours after start of doxycycline treatment, and viability assessed by trypan blue exclusion. n=3; mean ± SD; Student’s *t*-test (two-sided, equal variance).

To determine if PRMT1 and/or PRMT5 are required for the growth of ES cells, we conducted a dose response analysis into the effects of the specific PRMT5 inhibitor GSK591 (29, 30), the tool compound to the clinically relevant PRMT5 inhibitor GSK3326595 (35). To inhibit PRMT1 we used the Type I inhibitor MS023 that displays a strong preference towards the inhibition of PRMT1, with a 6-fold lower *in vivo* IC_50_ for PRMT6, the next best-targeted Type 1 PRMT enzyme (31). We found that a 4-day treatment of GSK591 or MS023 with low concentrations of compounds led to a dose-dependent reduction in cell proliferation in the *EWSR1::FLI1* positive fusion ES cell lines SK-NM-C, TC-32 and A673 (**Figures 2A–F**). TC-32 responded similarly to MS023 and GSK591 resulting in a 66.14% and 63.5% reduction in cell viability at the highest concentration of inhibitor (500nM of GSK591, 1μM MS023). In contrast, both SK-N-MC and A673 displayed increased sensitivity to GSK591. This was particularly striking in A673 where only 33.9% of cells were remaining after treatment with the low dose of 100nM GSK591. As expected, in all three cell lines, GSK591 reduced cellular SDMA but also led to a concurrent increase in ADMA in SK-N-MC cells. Likewise, MS023 reduced cellular ADMA levels but also increased SDMA and monomethylation (MMA) (**Figure 2G**, **Supplementary Figure 2**). The ability of PRMT5 and Type I inhibition to lead to increases in the alternative form of dimethylation is a well-documented phenomenon of substrate scavenging evident at global levels of ADMA/SDMA (36). Although MS023 has a higher efficacy for PRMT1 over other Type I enzymes (31), we wondered what proportion of the MS023 response was via PRMT1 inhibition. We therefore generated SK-N-MC cell lines with doxycycline-inducible knockdown of PRMT1 and found that depletion of PRMT1 led to a similar effect on cell growth as addition of MS023 (**Figure 2H**), strongly implying that the effects of MS023 are largely mediated by inhibition of PRMT1. Similarly, shRNA-mediated depletion of PRMT5 affected cell survival to a similar extent as GSK591 (**Figure 2I**).

### Combining type 1 inhibitor and GSK591 promotes apoptosis of ES cells but not in normal bone marrow mesenchymal stem cells

**Figure 3:**
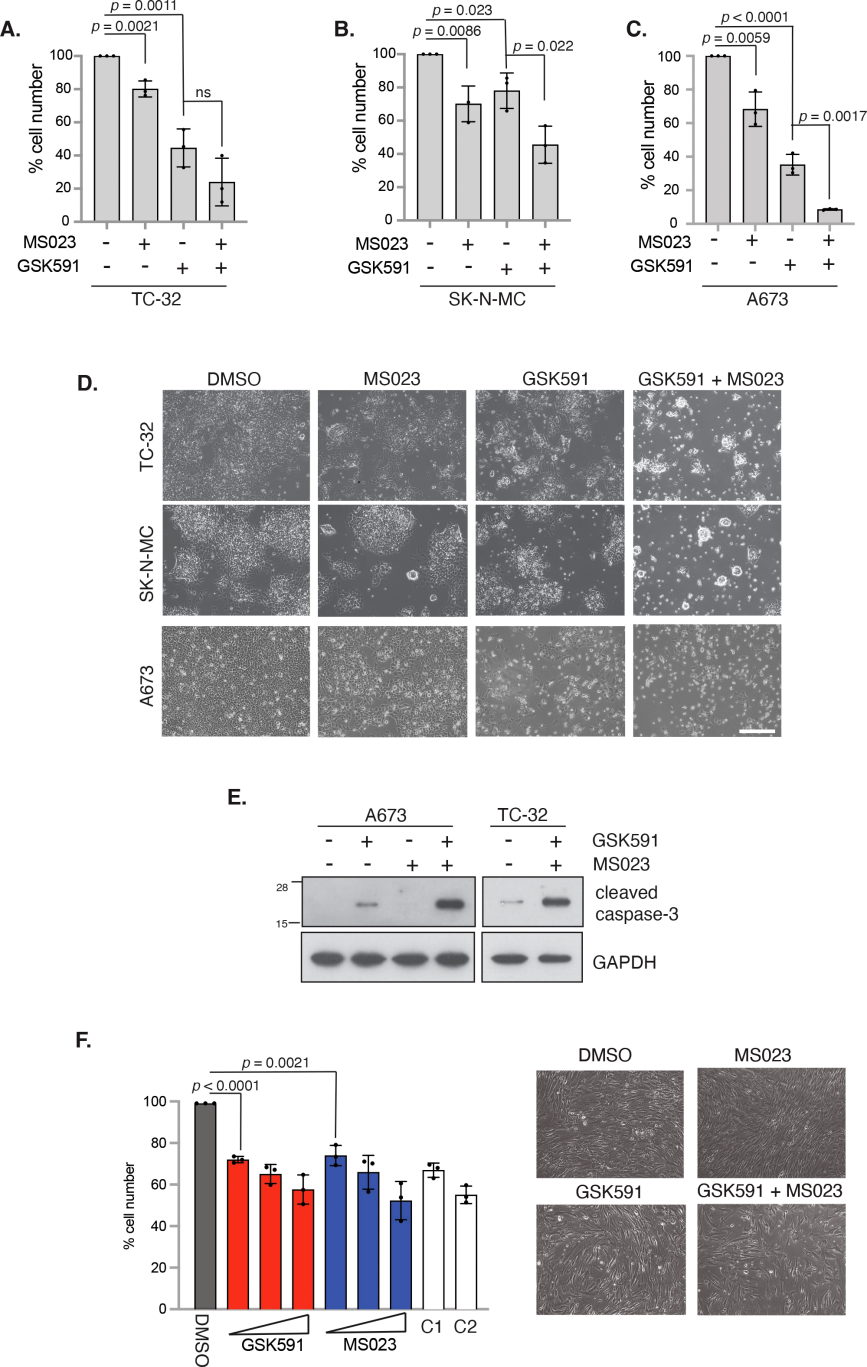
Combination PRMT5 and Type 1 PRMT inhibitors synergise to promote apoptosis in EWS-FLI1 cells. **(A)** TC-32 cells were treated with 100nM GSK591 or 100nM MS023 for 4 days, and viability assessed by trypan blue exclusion. ns, not significant. **(B)** SK-N-MC cells were treated with 250nM GSK591 or 250nM MS023 for 4 days, and viability assessed by trypan blue exclusion. **(C)** A673 cells were treated with 100nM GSK591 or 250nM MS023 for 4 days, and viability assessed by trypan blue exclusion. **(D)** Representative brightfield images of cells in A-C after 4 days treatment. Scale bar = 300nm. **(E)** Cells were treated with inhibitors for 4 days and apoptosis assessed by immunoblotting for cleaved caspase-3. **(F)** Bone marrow mesenchymal stem cells (BMMSCs) were treated with GSK591 (100nM, 250nM, 500nM), MS023 (100nM, 250nM, 1μM) or combination (C1 = 100nM GSK591/MS023; C2 = 250nM GSK591/MS023) for 5 days and viability assessed by trypan blue exclusion. n=3; mean ± SD; Student’s *t*-test (two-sided, equal variance). To the left of graph: Representative brightfield images of BMMSC after 5 days treatment of MS023 (250nM) and/or GSK591 (250nM). Scale bar = 300nm. In all panels, n=3; mean ± SD; Student’s *t*-test (two-sided, equal variance).

One concern for PRMT monotherapy treatment is the possibility that clinically relevant doses may produce adverse effects that limit clinical applications. Alternatively, combining low concentrations of PRMT1 or PRMT5 inhibitors is well tolerated in mice and can synergically reduce growth and promote apoptosis in pancreatic cancer and DLBCL cancer cells (37). Taking low concentrations of GSK591 and MS023 that exhibited partial growth suppression (SK-N-MC = 250nM for both inhibitors; TC-32 = 100nM for both inhibitors; A673 = 100nM for GSK591 and 250nM for MS023), we found that a 4-day treatment of both compounds promoted a greater growth inhibition effect than either monotherapy (**Figures 3A–D**). This was particularly striking in A673 cells with a 91.06% reduction in cell viability after combination treatment (**Figure 3C**). Indeed, combination GSK591 and MS023 appears to be promoting apoptosis in all three cell lines, supported by an induction of cleaved caspase-3 (**Figures 3D, E**). Interestingly, low dose GSK591 was also able to induce a low level of apoptosis as a monotherapy in A673 cells (**Figure 3E**).

We next wanted to determine the effects of MS023 and GSK591 on the growth of non-transformed BMMSC cells. Interestingly, we found that monotherapy drug treatments only led to a modest reduction in cell proliferation at the low doses that showed drastic effects in ES cells. For example, 100nM GSK591 or MS023 only lead to a 27.3% and 25.3% reduction in cell proliferation respectively. Moreover, the dose response effect for either MS023 or GSK591 observed in ES cells was largely absent in BMMSCs, and combination treatment of GSK591/MS023 did not further supress growth beyond that of either single agent at the same concentrations nor induced apoptosis (**Figure 3F**). Taken together, our data implies that GSK591 alone induced low level apoptosis, and that combining low dose Type 1/PRMT5 inhibitors drives a more potent apoptotic response in ES cells. Critically, the effect of either single agent or combined therapy on normal BMMSCs is minimally cytostatic thereby suggesting a therapeutic window of opportunity that could potentially mitigate clinically associated adverse effects.

### PRMT inhibitors synergise with olaparib inducing cell death and are dependent on *EWSR1::FLI1* expression for cytotoxic effect

**Figure 4:**
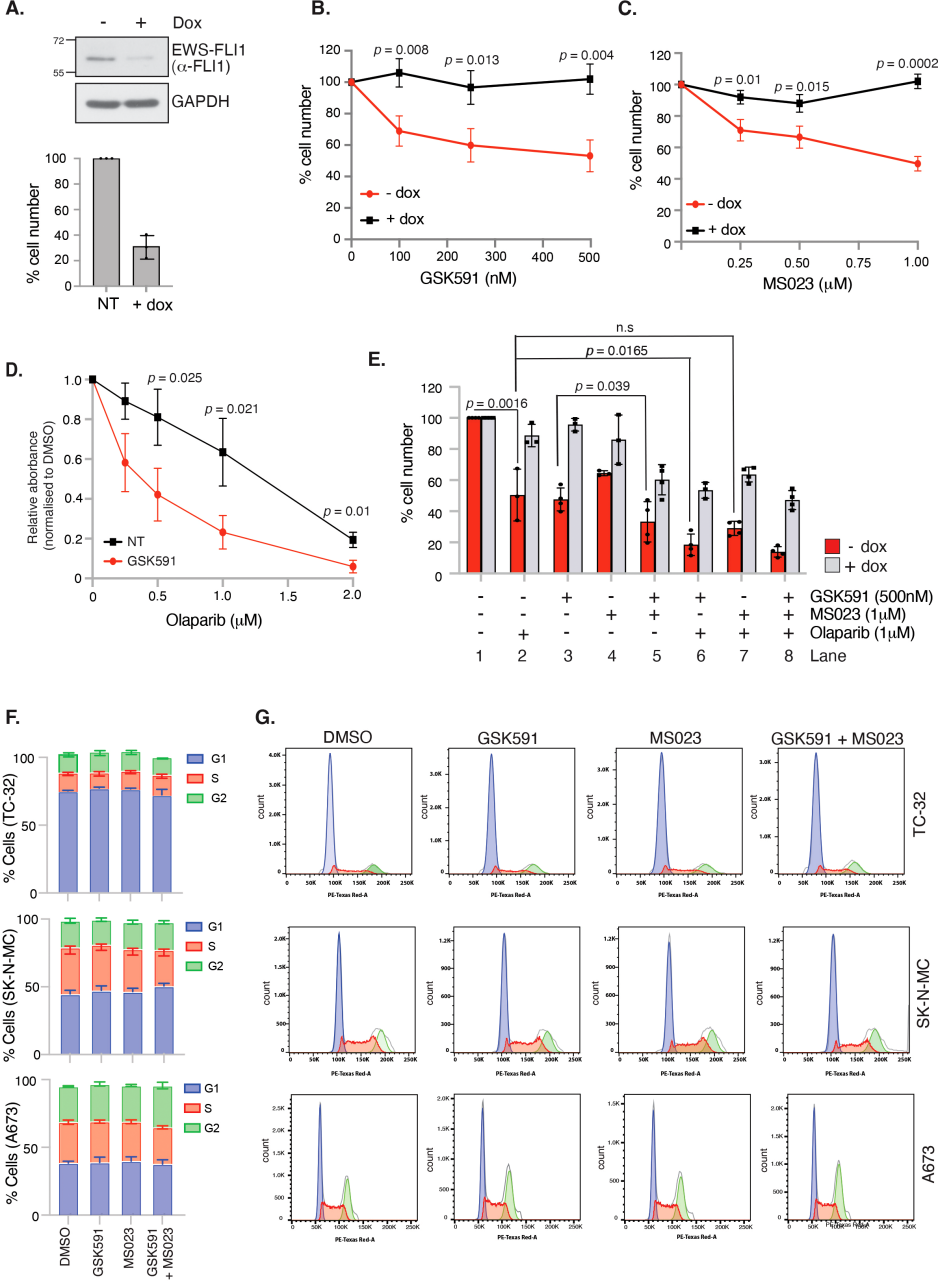
GSK591 and MS023 reduce A673 cell viability in a *EWSR1::FLI1*-dependent manner. **(A)** Immunoblot demonstrating inducible knockdown of *EWSR1::FLI1* in A673-tetON-EWSR1::FLI1 cells with corresponding decrease in cell viability, as measured by trypan blue exclusion. Cells were treated with 2μg/ml doxycycline for 7 days before analysis. n=3; mean ± SD. **(B)** Knockdown of *EWSR1::FLI1* suppresses GSK591-induced reduction in cell viability. A673-tetON-EWSR1::FLI1 cells were treated for 3 days with doxycycline before drug treatment with GSK591 for a further 4 days. Cell proliferation was measured by trypan blue exclusion assay. n=3; mean ± SD; Student’s *t*-test (two-sided, equal variance). **(C)** Knockdown of *EWSR1::FLI1* suppresses MS023-induced reduction in cell viability. A673-tetON-EWSR1::FLI1 cells were treated for 3 days with doxycycline before drug treating with MS023 for a further 4 days. n=3; mean ± SD; Student’s *t*-test (two-sided, equal variance). **(D)** GSK591 (125nM) A673 cells to olaparib. Cells were treated for 6 days and cell viability/metabolism measured by MTT assay. n=3; mean ± SD; Student’s *t*-test (two-sided, equal variance). **(E)** GSK591 synergises with olaparib to reduce viability that is partially dependent on *EWSR1::FLI1*. A673-tetON-EWSR1::FLI1 cells were treated for 3 days with doxycycline before drug treating with MS023 (1μM) and/or GSK591 (500nM) for a further 4 days. Olaparib (1μM) was administered for 7 days. Cell proliferation was measured by trypan blue exclusion assay. n>3; mean ± SD; Student’s *t*-test (two-sided, equal variance). ns, not significant. **(F)** 48 hrs GSK591, MS023 or combination does not affect cell cycle. TC-32 = 100nM GSK591/MS023; SK-N-MC = 250nM GSK591/MS023; A673 = 100nM GSK591; 250nM MS023. n=3; mean ± SD. **(G)** Representative cell cycle profiles of data presented in **(F)**.

The defining feature of 85-90% of ES cancers is expression of the EWS-FLI1 oncogenic fusion protein that is essential for cellular transformation and cancer growth (1, 38). Direct drug targeting of EWS-FLI1 is challenging given the lack of catalytic activity, hence pathways that are dependent on the function of EWS-FLI1 have been suggested as an alternative approach that could confer synthetic lethal and hence tumour-specific targeting. Firstly, we examined if EWS-FLI1 contributes to expression of PRMT family members by interrogating the ESCLA RNA-sequencing datasets derived from a panel of 18 ES cell lines that had undergone shRNA-mediated depletion of *EWSR1::FLI1* (33). We found that reducing *EWSR1::FLI1* expression did not affect transcript levels of PRMT family members or MEP50 (**Supplementary Figure 3A**). Likewise, neither GSK591 nor MS023 altered EWS-FLI1 protein levels (**Supplementary Figure 3B**). Next, we wanted to understand if the growth inhibition exhibited by GSK591 and/or MS023 treatment was dependent on the EWS-FLI1 oncogenic fusion protein. To achieve this, we focused on the engineered A673 cell line utilised in the ESCLA dataset (A673-tetON-shEWSR1::FLI1 (28)) that enables controllable suppression of the oncogenic fusion without a major compromise in cell viability (**Figure 4A**). Here, we found that the effects of single agent GSK591 and MS023 were largely supressed by partial depletion of *EWSR1::FLI1* (**Figures 4B, C**; **Figure 4E** Lane 3 and 4), however the reduced viability exhibited by combining GSK591/MS023 was only partially dependent on *ESWR1::FLI1* (**Figure 4E**, Lane 5). This data therefore indicates a functional link between PRMT1, PRMT5 and EWS-FLI1 that is important for ES cell survival, but that dual MS023/GSK591 therapy that suppresses most arginine methylation events in the cell reduces cell survival in both a fusion dependent and independent manner.

Next, we wanted to examine if PRMT inhibitors could synergise with olaparib in A673 cells. ES cells are genetically BRCA1 wildtype, however the ability of the EWS-FLI1 fusion protein to sequester BRCA1 promotes HR deficiency leading to olaparib sensitivity (6). Moreover, both PRMT1 and PRMT5 are known regulators of HR-mediated repair. For example, PRMT5 methylates RUVBL1 leading to 53BP1 mobilisation from double strand break ends and is required for the splicing of DNA repair genes (18, 39), whilst PRMT1-mediated methylation of MRE11 is enhanced in S/G2 phase of the cell cycle enabling nuclease-mediated DNA end resection that commits a cell to HR-mediated repair (24, 40). Treatment with low dose GSK591 led to a dose-dependent sensitisation of A673 cells to olaparib (**Figure 4D**). As expected, olaparib was only effective in reducing the survival of A673 cells when EWS-FLI1 was expressed (**Figure 4E**, Lane 2). Combination GSK591 and olaparib treatment was partially rescued by *EWSR1::FLI1* depletion (**Figure 4E**, Lane 6) implying that mechanisms of cytotoxicity with GSK591/olaparib involve both fusion dependent and independent events. Interestingly, we found that combining olaparib with MS023 did not lead to a significant reduction in cell viability (**Figure 4E**; compare Lane 2 and 7), suggesting that in ES cells, SDMA but not PRMT1-dependent ADMA contributes to DNA repair after olaparib-induced damage. One explanation for a lack of additive effect between MS023 and olaparib is the potential for MS023 to lead to a G1 cell cycle arrest, thereby reducing the number of cells that can enter S-phase and encounter olaparib-induced damage (41). However, 48 hrs treatment of single agent MS023 or GSK591, or combination MS023/GSK591 treatment, did not affect the cell cycle nor the ability of A673 cells to enter S-phase and undergo DNA synthesis (**Figures 4F, G**, **Supplementary Figure 4**). Taken together, our data suggests that combining PRMT5 inhibitors with agents that require DNA replication for cytotoxicity is an interesting new avenue for the treatment of ES.

### Arginine methylation regulates genome stability in ES cells

**Figure 5:**
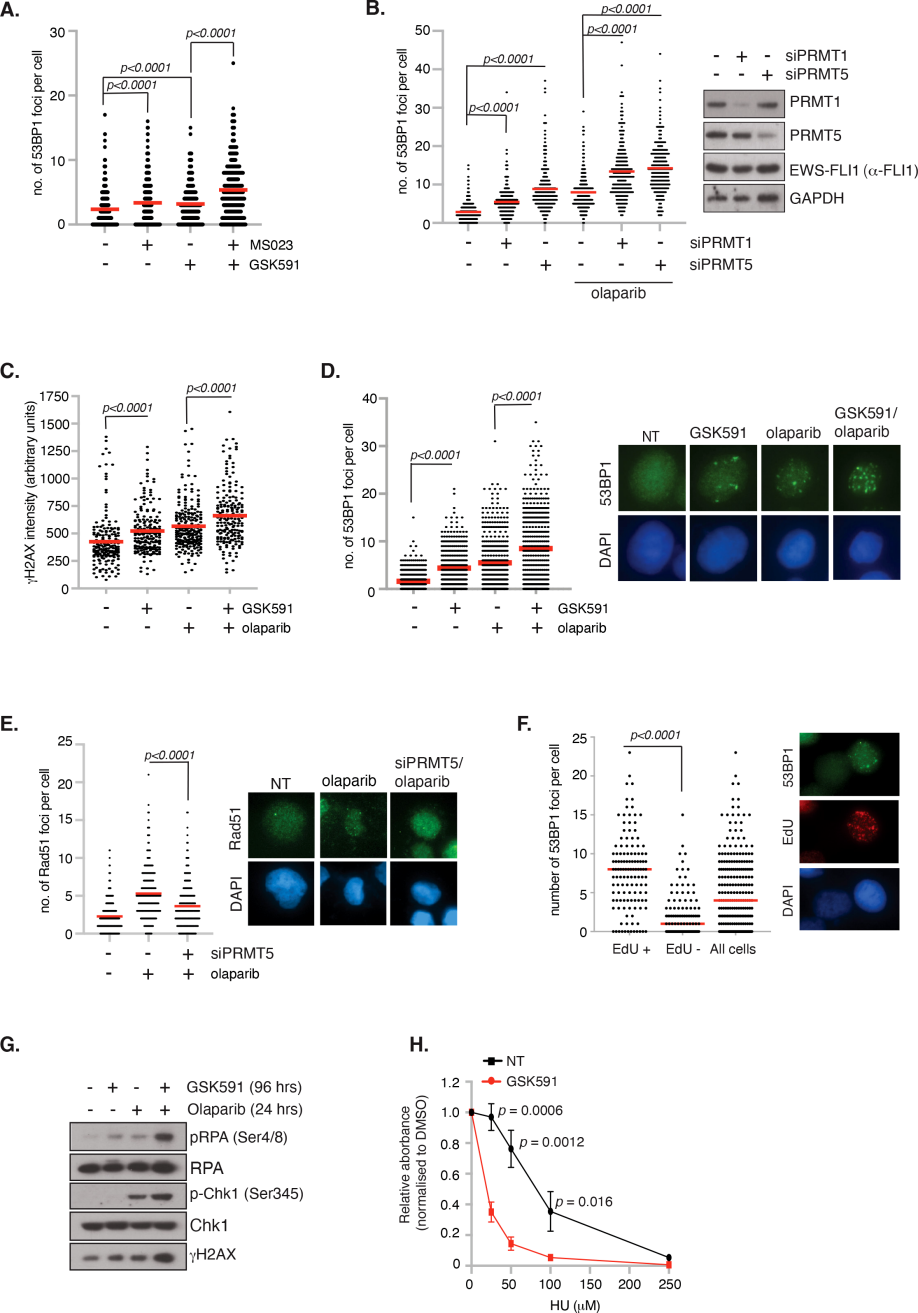
Inhibition of PRMT5 promotes replication stress and DNA damage. **(A)** TC-32 cells were treated with 100nM GSK591, MS023 or in combination for 24 hrs and the number of 53BP1 foci per cell quantified. At least 100 cells per biological replicate were analysed. n=3; mean ± SD; Student’s *t*-test (two-sided, equal variance). **(B)** Depletion of PRMT1 and PRMT5 in A673 cells induces 53BP1 foci formation and augments olaparib (1μM; 24 hrs)-induced damage. At least 75 cells per biological replicate were analysed. n=3; mean; Student’s *t*-test (two-sided, equal variance). **(C, D)** GSK591 synergises with olaparib inducing **(C)** γH2AX and **(D)** 53BP1 foci. A673 were pretreated with GSK591 (500nM) for 72 hrs or olaparib (1μM) for 24 hrs. For combination treatments, cells were pretreated with GSK591 for 48 hrs before addition of olaparib for a further 24 hrs. At least 100 cells per biological replicate were analysed. n=3; Student’s *t*-test (two-sided, equal variance). Representative images are to the right of data. **(E)** Depletion of PRMT5 in A673 cells supresses olaparib-induced Rad51 foci formation. At least 75 cells per biological replicate were analysed. n=3; mean; Student’s *t*-test (two-sided, equal variance). Representative images are to the right of the data. **(F)** DNA damage induced by PRMT5 inhibition in A673 cells is enriched in EdU+ S-phase cells. Cells were treated with GSK591 (500nM) for 48 hrs and the number of 53BP1 foci per cell analysed. n=2; mean; Student’s *t*-test (two-sided, equal variance). Representative images are to the right of the data. **(G)** Combination GSK591 (500nM; 4 days) and olaparib (5μM; 24 hrs) induces a replication stress response as indicated by increased phosphorylation of RPA and Chk1. Immunoblot is representative of 3 biological experiments. **(H)** GSK591 (125nM) sensitises A673 cells to hydroxyurea (HU). Cells were treated for 6 days and cell viability/metabolism measure by MTT assay. n=3; mean ± SD; Student’s *t*-test (two-sided, equal variance).

Having demonstrated that PRMT inhibitors lead to reduced cell viability and apoptosis, we next wanted to determine if this was due to increased DNA damage leading to genome instability. We therefore treated TC-32 cells with low dose GSK591 and MS023 and quantified the number of DSBs as indicated by 53BP1 foci formation 24 hrs after drug treatment, hence capturing the early events before cell death. Monotherapy of MS023 or GSK591 resulted in a significant amount of DNA damage in the absence of exogenous stressors, which was significantly exacerbated by combining GSK591 with MS023 (**Figure 5A**). Likewise, depletion of PRMT1 or PRMT5 in A673 cells led to elevated DNA damage as determined by 53BP1 foci formation (**Figure 5B**; **Supplementary Figure 5**). Focusing on the effects of GSK591, either alone, or in combination with olaparib, we found that GSK591 monotherapy resulted in a significant amount of DNA damage in A673 cells, as determined by γH2AX intensity and 53BP1 foci formation. Combining GSK591 with olaparib, or depleting PRMT5 with siRNA, induced more DNA damage than either agent alone (**Figures 5B–D**) suggesting that the reduced viability is in part due to a defective DNA damage response. Indeed, depletion of PRMT5 reduced olaparib-induced RAD51 foci formation implying defective HR (**Figure 5E**). Interestingly, we noticed that GSK591 appeared to induce two populations of cells with one group experiencing relatively high levels of damage per cell. Given that the main mechanism of olaparib cytotoxicity is through the trapping of PARP leading to DNA replication interference and replication stress (42), we posit that GSK591 may be further promoting replication stress and therefore be a more potent inducer of DNA damage in S-phase cells. Supporting this, we found that GSK591-induced DSBs were enriched in EdU+ S-phase cells (**Figure 5F**) and that levels of RPA phosphorylation (Ser4/8), a marker of RS-induced checkpoint activation and regulator of replication for restart and late origin firing (43), were similar to that induced by single agent olaparib treatment (**Figure 5G**). As such, combination GSK591 and olaparib greatly increased RPA and Chk1 phosphorylation (**Figure 5G**). Moreover, GSK591 also sensitised A673 cells to the replication stress-inducing agent hydroxyurea (**Figure 5H**). Taken together, our data suggests that PRMT5 is required for the replication stress response in ES and that inhibition leads to accumulation of DNA damage during S-phase that manifests to a cytotoxic level over time and predisposes cells to additional replication-inducing agents such as PARP inhibitors.

## Discussion

In this study, we identify a role for the arginine methyltransferases PRMT1 and PRMT5 in the survival of Ewing sarcoma cells. We find that PRMT1 and PRMT5 expression is elevated in a panel of ES cell lines and regulates genome stability because inhibition of either PRMT1 or PRMT5 resulted in DNA damage and DSB induction that was significantly exacerbated by combining both PRMT inhibitors. Mechanistically, PRMT5 appears to be involved in the replication stress response because GSK591 preferentially led to DNA damage in S-phase cells and can potentiate the effects of replication stress-inducing agents olaparib and HU promoting cell death. Interestingly, BMMSCs representative of normal tissue only experienced modest growth inhibition after combination of both inhibitors. This, coupled with our findings that the effects of PRMT inhibitors depend on the expression of *EWSR1::FLI1*, indicate that drug treating ES patients with PRMT inhibitors, with or without PARP inhibitors, offers a potential new therapeutic approach.

Clinical inhibition of PRMT activity has gained significant interest in the treatment of adult solid cancers and haematological disease with PRMT5 inhibitors rapidly entering phase I/II clinical trials (22). Whereas PRMT1 inhibitors struggled in Phase I with no inhibitor currently in clinical development, first-generation PRMT5 inhibitors have demonstrated encouraging clinical activity with good target engagement and manageable adverse effects (44–46). However, predictive biomarkers for response are lacking and have thus limited the drug development of these first-generation PRMT5 inhibitors. Our *in vitro* analysis using a panel of ES cell lines treated with the PRMT5 tool compound GSK591 are therefore particularly encouraging for the treatment of ES patients. Given that the effect of PRMT5 inhibition is largely dependent on the presence of the ES-specific fusion, we hypothesise that this fusion-induced sensitivity to PRMT5 inhibition provides a unique therapeutic window compared to normal tissues. As such, the fusion itself could potentially serve as predictive biomarker for responses to first-generation PRMT5 inhibitors.

ES are highly aggressive cancers and GSK591 demonstrated synergy with olaparib and HU. As such, rather than proposing single agent PRMT5 inhibitor treatment, a combination treatment with a next generation PARP1 specific inhibitor that displays less haematologic toxicity, e.g. AZD5305 (47) could be promising. Our data thus supports further studies evaluating the *in vivo* efficacy of first-generation PRMT5 inhibitors in combination with PARP inhibitors in preclinical ES PDX models, as well as more detailed mechanistic analysis and potential biomarker development beyond the EWS-FLI1 fusion protein. Interestingly, although ES is a cancer of low mutational burden, 12% of patients have *CDKN2A* deletion (48) an event that often leads to co-deletion of *MTAP.* MTAP is an enzyme that is part of the methionine salvage pathway that metabolically converts methylthioadenosine (MTA) to methylthioribose (MTR) and adenine ultimately leading to methionine regeneration. MTA was shown to biochemically compete with SAM for PRMT5 binding leading to a moderately potent but selective inhibition (49–51). Consequently, MTAP deleted cancers are particularly vulnerable to further inhibition with second-generation PRMT5 inhibitors that can selectively target the PRMT5:MTA complex leading to a synthetic lethal relationship (52, 53). As these second-generation inhibitors are also in Phase I development for adult cancers [NCT06672523; NCT063690354; NCT06137144; NCT06130553; NCT06589596], including adult sarcoma [NCT05732831], a clinical trial with one of these inhibitors in ES patients with *MTAP* deletion could also be considered after further preclinical *in vitro* and *in vivo* assessment. Together, our data using the PRMT5 inhibitor GSK591 has the potential to rapidly expedite clinical trial development in paediatric and young adult ES patients using these compounds.

Whilst many of the identified oncogenic functions of *EWSR1::FLI1* are known functions of PRMTs, the detailed mechanisms by which PRMTs regulate the survival of *EWSR1::FLI1* ES cells is still largely unknown. ES cells experience a high degree of replication stress and R-loop formation due to EWS-FLI1-dependent promotion of CDK9-mediated RNA Polymerase II activation (6, 54). To counteract this, ES cells upregulate proteins that are part of the normal replication stress response providing a buffering system to ensure cancer cell survival. Consequently, treatment of ES cells with drugs that inhibit essential components of the replication stress response, e,g. Chk1, ATR and CDC7 (54–57), or further exacerbate replication stress [e.g. replication inhibiting agents aphidicolin, clofarabine and PARP inhibitors (6, 56)] induced DNA damage and cell death and are being explored clinically in ES patients. For example, ES patients with classical *EWSR1* fusions have been included in the ESMART (European Proof of Concept Therapeutic Stratification Trial of Molecular Anomalies in Relapsed or Refractory Tumors in Children and Adolescents) trial (58, 59) comprising of Arm D [combination irinotecan and olaparib (12)] and Arm N [combination olaparib and ATR inhibitor ceralasertib (60)]. Likewise, a Phase II randomised trial of the PARP inhibitor talazoparib with liposomal irinotecan vs temozolomide with liposomal irinotecan is currently being undertaken in relapsed/refractory ES patients [NCT04901702], whilst a single arm Phase II study of trabectidine with low dose irinotecan appears promising [NCT04067115] (61). Our findings that PRMT5 is upregulated in ES cells contributing to genome stability and DNA repair after replication stress is highly suggestive that PRMT5 is an unappreciated component of the ES cell replication stress buffering system counteracting deleterious *EWSR::FLI1*-induced replication stress. This finding allows us to consider PRMT5 inhibitors as an additional and potentially more targeted class of drugs to treat ES. Future studies that understand the contributions of PRMT5 to the replication stress response in ES cells will shed new light into the mechanisms of malignant progression and resistance development that could be further exploited therapeutically.

## Data Availability

The original contributions presented in the study are included in the article/**Supplementary Material**. Further inquiries can be directed to the corresponding authors.

## Supplementary material

Supplementary Figure 1 Transcript expression of *PRMT1, PRMT5* and *MEP50* across a panel of Ewing Sarcoma, breast and lung cancer tissue datasets of patients. Data taken from the R2 Affymetrix platform (MAS 5.0-u133p2- *PRMT5* 217786_s_at; *MEP50/WDR77* 201421_s_at; *PRMT1* 206445_s_at).

Supplementary Figure 2 Dose effects of GSK591 (0-500nM) and MS023 (0-1μM) on SDMA, ADMA and MMA levels in TC-32 and A673 cells. Representative image of n=3 independent experiments.

Supplementary Figure 3
**(A)** mRNA expression levels of *PRMT1, PRMT2, CARM1, PRMT5, PRMT6, PRMT7, PRMT9 and MEP50* after inducible knockdown of *EWSR::FLI1* in a panel of ES-tetONE-EWSR1::FLI1 cell lines. Data is normalised to non-induced control. Cell lines used in our study are highlighted blue. Data taken from the ESCLA dataset (33). **(B)** A673 cells were treated with GSK591 (500nM) or MS023 (1μM) for 3 days and immunoblotted as indicated. Representative image of n=2.

Supplementary Figure 4 48 hrs GSK591, MS023 and combination does not alter entry into S-phase in A673 cells (100nM GSK591; 250nM MS023). n=3; mean ± SD.

Supplementary Figure 5 Representative images of data presented in **Figure 5B**. Scale bar = 10nm.
