# Supplementary figures and images for: Arginine methylation regulates Ewing sarcoma cell viability in a EWSR1::FLI1 dependent manner and provides a therapeutic opportunity
Source: Front Oncol. 2025 Aug 1;15:1538208. doi: 10.3389/fonc.2025.1538208 (PMC12354397; doi:10.3389/fonc.2025.1538208)

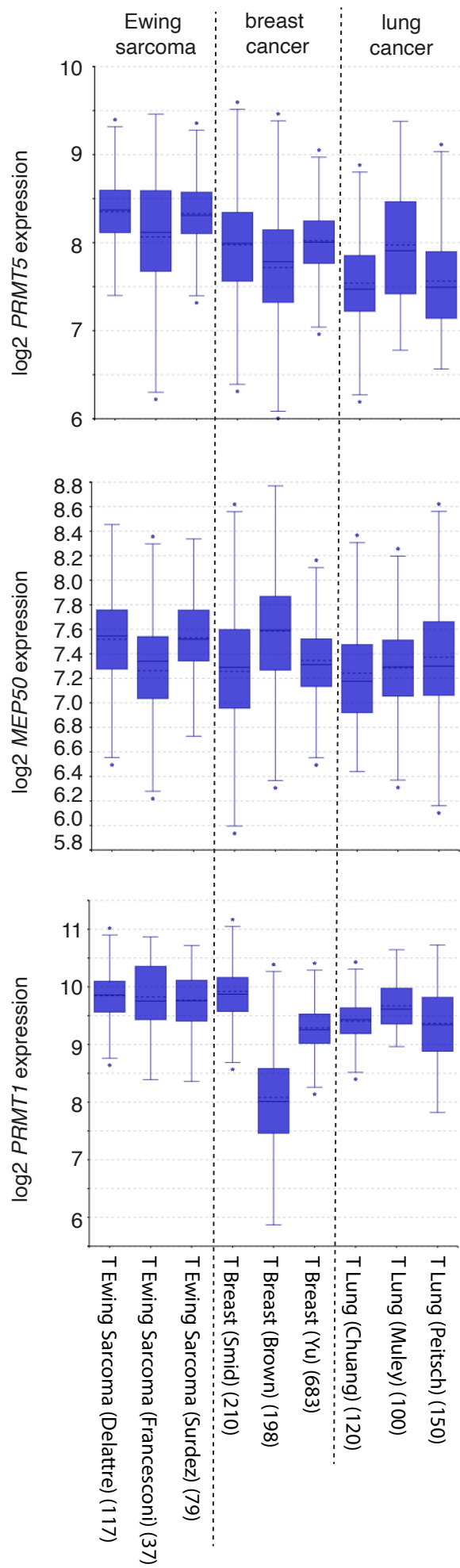

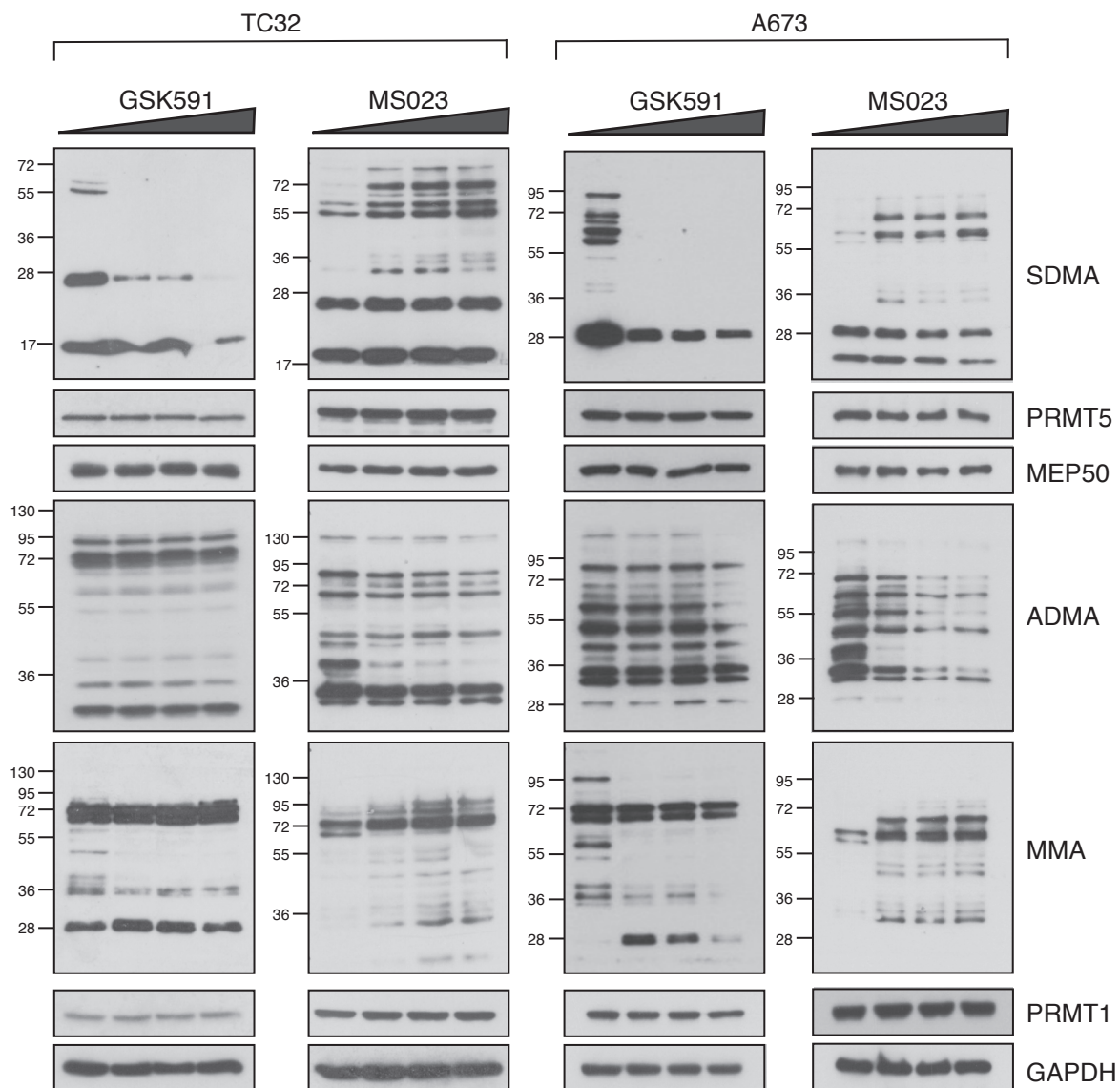

A.

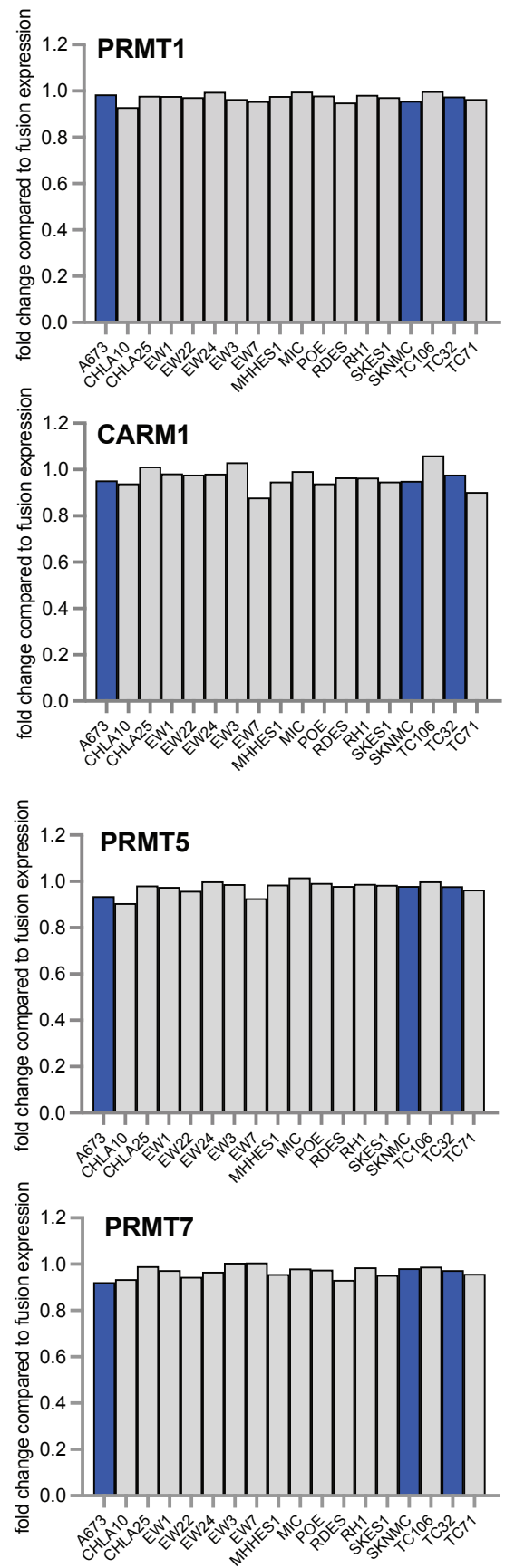

B.

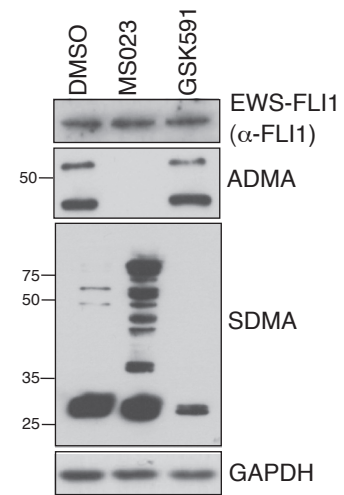

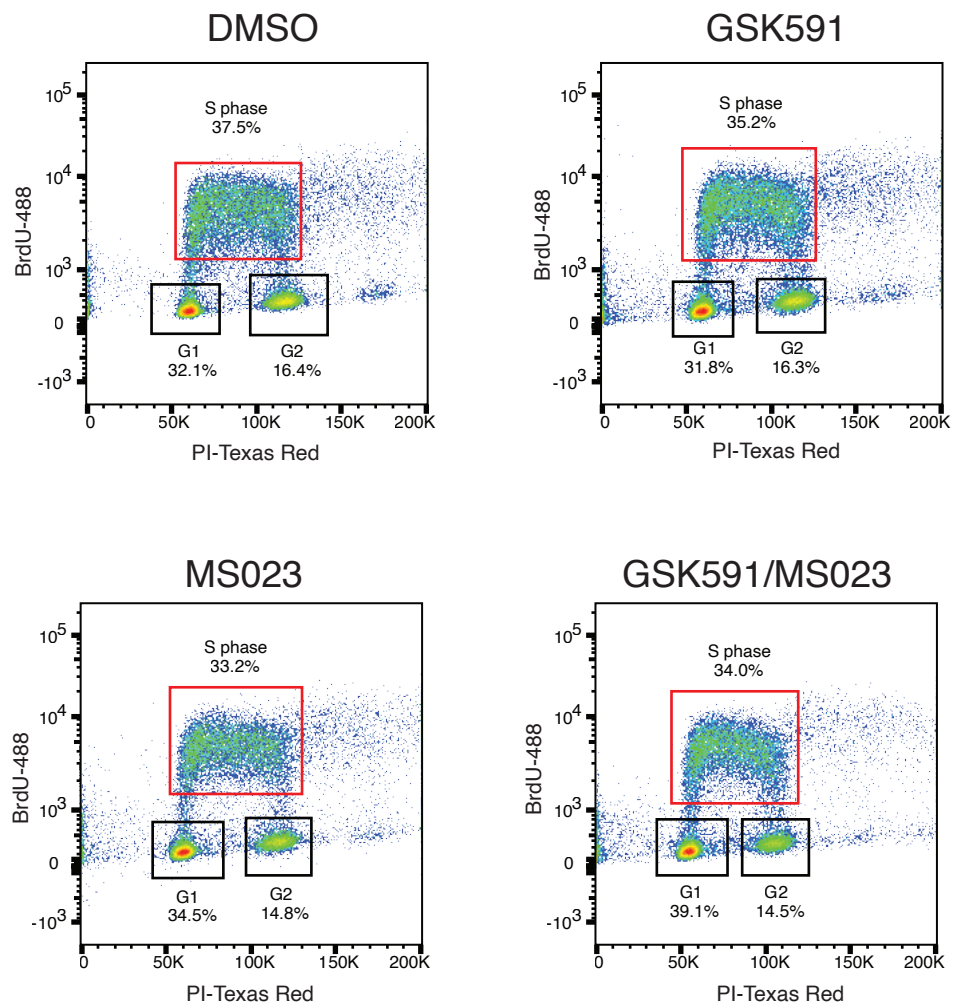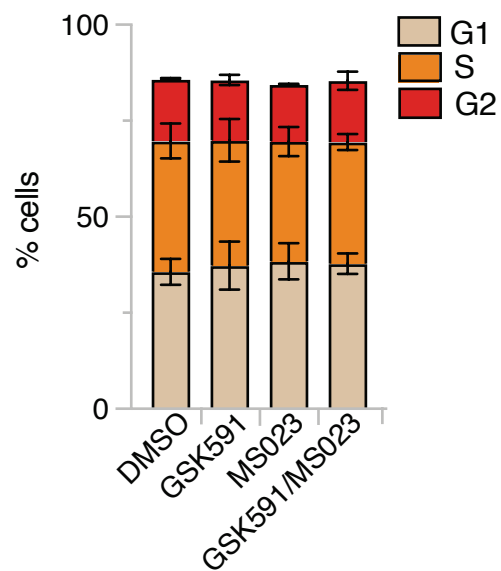

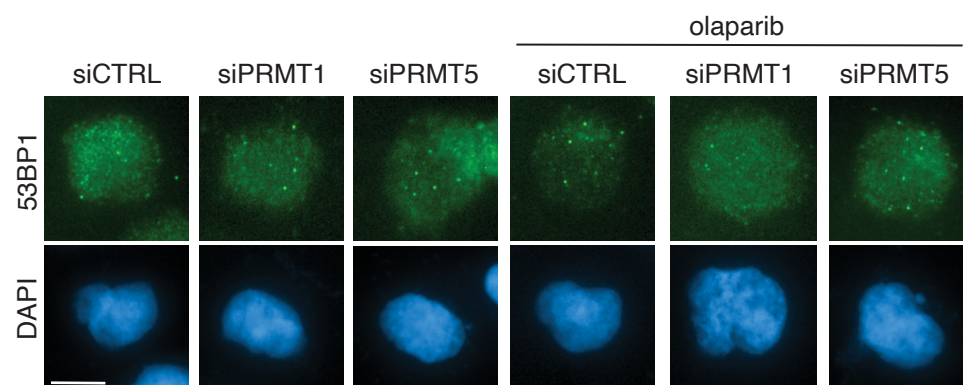

Supplement: Supplementary Figure 1 — Transcript expression of PRMT1, PRMT5 and MEP50 across a panel of Ewing Sarcoma, breast and lung cancer tissue datasets of patients. Data taken from the R2 Affymetrix platform (MAS 5.0-u133p2- PRMT5 217786_s_at; MEP50/WDR77 201421_s_at; PRMT1 206445_s_at). [file DataSheet1.pdf]
